# Factors influencing the healthcare transition in Chinese adolescents with inflammatory bowel disease: a multi-perspective qualitative study

**DOI:** 10.1186/s12876-023-03080-z

**Published:** 2023-12-18

**Authors:** Mi Zhou, Youjun Xu, Yunxian Zhou

**Affiliations:** School of Nursing, Zhejiang Chinese Medical University, 548 Binwen Road, Binjiang District, Hangzhou, Zhejiang 310053 China

**Keywords:** Inflammatory bowel Disease, Pediatric, Adolescent, Transition, Healthcare

## Abstract

**Background:**

The development and implementation of the transition from pediatric to adult healthcare systems for adolescents with inflammatory bowel disease (IBD) should consider stakeholders’ perceptions. This study aimed to explore the factors influencing the transition of Chinese adolescents with IBD from the perspectives of patients, parents, and healthcare providers.

**Methods:**

A descriptive qualitative research was conducted. Purposive sampling was used to recruit 36 participants, including 13 patients, 13 parents, and 10 providers, from a tertiary pediatric IBD center, a tertiary adult IBD center, and the China Crohn’s & Colitis Foundation in Zhejiang Province, China. Individual semi-structured interviews were used to collect data on facilitators and barriers to the transition process. Conventional content analysis was used to analyze the interview transcripts.

**Results:**

Nine primary themes were identified. Patients with young age, prolonged disease duration, severe disease, academic pressures such as the Gaokao, low level of disease acceptance, limited transition consciousness, low self-efficacy, poor transition communication, and inadequate medical transition system serve as barriers. While patients with the mentality of guilt towards their parents; parents with low education levels and intensive work schedules, high levels of disease acceptance, and situations of parent-child separation; stakeholders with high transition consciousness, high transition self-efficacy, and effective transition communication act as facilitators. Furthermore, community support and hospital guide services were also contributing factors during the transition.

**Conclusions:**

This study offers comprehensive insights into the factors affecting the transition of Chinese adolescent IBD patients. The process is continuously influenced by stakeholders, community, and healthcare environments and policies. Identifying these factors provides healthcare providers with a reference for developing and implementing targeted transition interventions.

## Background

Inflammatory bowel disease (IBD), including Crohn’s disease (CD) and ulcerative colitis (UC), is a chronic autoimmune disorder impacting the gastrointestinal tract. The incidence of IBD among children and adolescents is rising globally [1, 2]. This subgroup typically exhibits more aggressive disease, higher rates of non-adherence, and a reduced time to surgery compared to adult [3–5]. The clinical severity of IBD significantly affects the growth, bone health, and pubertal development in this young demographic [6]. Moreover, adolescents with IBD encounter greater challenges when transitioning to higher education and experience elevated stress levels relative to their healthy peers [7, 8]. There are some differences between pediatric and adult care models. Pediatric care typically adopts a family-centered, multidisciplinary, and comprehensive approach, while adult care tends to be patient-centered, and advocates patient independence and self-management [9, 10]. As adolescents with IBD mature, the transition from pediatric to adult healthcare systems becomes inevitable.

Transition refers to the purposeful and planned transfer of individuals with chronic physical and medical conditions from the pediatric to adult healthcare systems [11]. The smooth transition of adolescents with chronic diseases has been widely identified as a national priority [12–14]. Nevertheless, studies indicate that adolescents with IBD are more susceptible during this transition and often experience difficulties [15, 16]. Furthermore, several healthcare providers overestimate these patients’ readiness for transition [17]. Poor managed transition is associated with adverse health outcomes [18], while planned transition for adolescents with IBD has been shown to promote remission [19]. Presently, there is no optimal model to guide clinical healthcare professionals in facilitating the IBD transition process [9], and this is also the case in China. The Chinese pediatric healthcare systems typically serve IBD patients up to the age of 18, whereas adult healthcare systems services commence at 14 years old. This leaves a gap for those aged 14–18, who must choose between systems without a formal transition program. The decision of whether, when, and where to transition largely relies on personal and parental preferences rather than systematic referral support from the healthcare system.

The Social-ecological Model of Adolescents and Young Adults Readiness to Transition (SMART) was created by Schwartz and colleagues a decade ago [20]. This model acknowledges the multi-systemic and social-ecological nature of transition, encompassing components such as sociodemographic/culture, access/insurance, medical status and risk, neurocognition/IQ, knowledge, skills/self-efficacy, beliefs/expectations, developmental maturity, goals/motivation, relationships/communication, and psychosocial/emotions [20, 21]. Since its creation, the SMART has been widely applied as a framework for transitioning children with chronic diseases, including cancer and spina bifida patients [20, 21]. However, the applicability and validation of the model’s components specifically for the transition of adolescents with IBD remain uncertain due to its limited use in IBD care. Furthermore, different countries and states may have different factors affecting the transition owing to national conditions and cultural histories, thus these transition models of chronic disease may not be entirely applicable to the Chinese population. The body of research on transition interventions for adolescents with IBD is scarce, and predominantly reliant on expert opinion and consensus rather than on empirical evidence [9]. Transition is a collaborative endeavor that requires the involvement of multiple stakeholders [20]. Therefore, this study aimed to explore the perceptions and attitudes of three key groups (patients, parents, and pediatric and adult healthcare providers) regarding the transition process in IBD and to identify the influencing factors. This study can provide a reference for healthcare providers to formulate and implement targeted transitional interventions.

## Methods

### Study design

This study used naturalistic inquiry to address the following research question: “What are the influencing factors of the transition for Chinese adolescent patients with IBD?” Naturalistic inquiry involves observing events in their natural settings without manipulating variables [22, 23]. Consistent with this approach, a multi-perspective, descriptive qualitative study was determined to be the most appropriate methodology [23]. The Standards for Reporting Qualitative Research (SRQR) were adhered to report this study [24].

### Participants

Purposive sampling was used to recruit participants from three stakeholder groups (patients, parents, and healthcare providers) [20]. Recruitment occurred from September 2020 to August 2021 at a tertiary pediatric IBD center, a tertiary adult IBD center, and the China Crohn’s & Colitis Foundation in Hangzhou City, Zhejiang Province, China. Researcher (XYJ) fostered initial rapport with potential participants through engaging in IBD-related volunteer activities, provided a verbal explanation of the study’s objectives and methods, and extended an invitation to participate. Snowball sampling was also used to expand recruitment.

Patients and their parents were invited to join the study as paired participants. Inclusion criteria for patients were: (a) definitive IBD diagnosis, (b) diagnosis before 18 years of age with a disease duration of at least three months, (c) age between 10 and 25 years, (d) undergoing or having completed the transition, and (e) ability to articulate themselves. We excluded patients who (a) had other chronic or underlying diseases that could affect the transition and (b) had cognitive impairment or psychiatric disorders. For parent participants, we included family caregivers whose children met the above criteria, as well as those who were articulating. Parents with cognitive impairment or psychiatric disorders were excluded. For provider participants, we included those who had (a) experience in treating or caring for over 100 patients with IBD and (b) at least one year of medical or nursing experience in the IBD specialty. Consent was obtained from the patients, parents, and providers before the interviews began. The sample size was based on the thematic saturation of each population profile [25].

### Data collection

We conducted one-to-one semi-structured interviews with participants from each population to explore in-depth perceptions and experiences of transition. All interviews were conducted by a female researcher, XYJ, who had completed training in qualitative research. Researcher ZYX, a female qualitative research expert with a Ph.D. from Australia and multiple published qualitative studies, oversaw the quality and integrity of the research process.

After a literature review and group discussion, the research team (ZM, XYJ and ZYX) created an initial draft of the interview outline for each stakeholder group. This outline was refined into a final interview guide following two pilot interviews per stakeholder group.

Before the formal interview, the interviewer established rapport with the interviewees, explained the topics and purpose of the interview, and obtained consent for audio recording. During the interviews, the interviewer listened to the participants, made detailed inquiries, clarified any confusion over time, and avoided leading questions. For example, when an adolescent patient stated, “I feel that my sense of independence is not strong, and I may need some guidance.” The interviewer asked, “Please tell me more about what guidance entails.” Additionally, nonverbal information from participants, such as facial expressions and body language, was observed. The interviews ended when participants indicated that they had no more to add. Transcriptions were completed within 24 h, and participants were given the transcripts for review and feedback.

The interviews were conducted in quiet and undisturbed homes (n = 6), clinics (n = 15), and a community location (n = 1) based on the participants’ preferences. Additionally, three telephone interviews and 11 video call interviews were conducted during the COVID-19 pandemic. The interview lengths ranged from 30 to 72 min, averaging 50 min for patients, 51 min for parents, and 42 min for providers.

### Data analysis

Conventional content analysis was used to analyze the qualitative data by coders ZM and XYJ [26]. First, ZM and XYJ read the interview transcripts repeatedly and gained a holistic understanding of them. Second, they manually coded the text line-by-line into condensed meaning units and inductively identified and compared concepts related to each stakeholder’s perspective on transition. They grouped similar and recurring codes into themes and subthemes. ZM and XYJ analyzed and coded the transcripts independently and then compared and consolidated their findings. The research team regularly reviewed the coded data, identified relationships among themes, deliberated on the implications of emergent findings, and agreed on the final themes. Saturation was deemed reached when new data ceased to contribute additional insights [27].

## Results

The study included 36 participants, consisting of 13 adolescent patients, 13 parents (matching the number of patients), and 10 healthcare providers, coded as A1-A13, B1-B13, and C1-C10 respectively. The demographic characteristics of each group are displayed in Table 1, 2 and 3. The qualitative analysis identified nine themes: personal characteristics, disease acceptance, transition consciousness, the mentality of guilt, self-efficacy, parent-child separation, transition communication, community support, and healthcare environment and policies (see Table 4). Among them, patients with young age, prolonged disease duration, severe disease, academic pressures such as the Gaokao, low level of disease acceptance, limited transition consciousness, low self-efficacy, poor transition communication, and inadequate medical transition system serve as barriers. While patients with the mentality of guilt towards their parents; parents with low education levels and intensive work schedules, high levels of disease acceptance, and situations of parent-child separation; stakeholders with high transition consciousness, high transition self-efficacy, and effective transition communication act as facilitators. Furthermore, community support and hospital guide services were also contributing factors during the transition.

**Table 1:** Demographic characteristics of adolescent patients (n = 13)

NO.	Gender	Age	Education Level	Registered permanent residence	Types of disease	Duration (month)	Surgery history	Disease activity
A1	Male	22	University	Country	CD	60	Perianal abscess resection	0a
A2	Female	16	High	Country	CD	7	/	3a
A3	Female	16	High	Country	CD	10	Small intestine resection	0a
A4	Female	17	High	Country	CD	7	Small intestine resection	1a
A5	Female	18	High	Town	CD	69	/	0a
A6	Male	13	Secondary	City	CD	26	/	0a
A7	Female	20	University	Town	UC	144	/	3b
A8	Male	19	University	City	UC	22	/	2b
A9	Female	19	University	City	UC	27	/	2b
A10	Male	19	University	Country	UC	61	/	2b
A11	Male	13	Secondary	Country	UC	16	/	6b
A12	Male	10	Primary	Country	UC	50	/	3b
A13	Female	17	High	City	CD	70	Partial obstruction resection	3a

a: The Harvey-Bradshaw index as CD activity indicators; b: the Walmsley index as UC activity indicators

**Table 2:** Demographic characteristics of parents (n = 13)

No.	Relationship	Age	Education Level	Marital status	Monthly personal income (CNY)	Occupation	Number of children
B1	Mother	45	Secondary	Married	<4000	Service industry	2
B2	Mother	50	Secondary	Married	<4000	Logistics	2
B3	Father	48	Secondary	Married	<4000	Laborer	2
B4	Mother	41	Secondary	Married	4000–8000	Logistics	2
B5	Mother	46	High	Married	4000–8000	Farmer	2
B6	Mother	37	Undergraduate	Married	8001–12,000	Financial industry	2
B7	Mother	46	Secondary	Divorce	<4000	Support industry	2
B8	Mother	58	High	Married	8001–12,000	Retiree	2
B9	Father	47	High	Married	4000–8000	Freelancer	2
B10	Mother	47	High	Divorce	<4000	Entrepreneurs	1
B11	Father	43	Secondary	Married	4000–8000	Entrepreneurs	3
B12	Mother	44	Secondary	Married	4000–8000	Entrepreneurs	2
B13	Mother	52	Undergraduate	Married	8001–12,000	Civil servant	2

**Table 3:** Demographic characteristics of health care providers (n = 10)

NO.	Occupation	Age	Education Level	Professional title	Length of work (years)	Length of treating or caring for IBD patients (years)	Number of treating or caring for IBD patients
C1	A nurse in the adult care system	29	Master	Primary nurse	<5	<5	301–400
C2	A physician in the adult care system	36	Master	Attending physician	5–10	<5	201–300
C3	A physician in the adult care system	45	Master	Associate chief physician	>15	>15	>400
C4	A physician in the adult care system	36	Doctor	Associate chief physician	5–10	5–10	201–300
C5	A physician in the adult care system	43	Master	Associate chief physician	>15	<5	101–200
C6	A nurse in the pediatric care system	37	Master	Associate chief nurse	11–15	5–10	101–200
C7	A nurse in the adult care system	48	Master	Associate chief nurse	>15	>15	>400
C8	A nurse in the pediatric care system	30	Undergraduate	Primary nurse	5–10	<5	>400
C9	A physician in the pediatric care system	40	Doctor	Associate chief physician	11–15	11–15	201–300
C10	A physician in the pediatric care system	39	Doctor	Associate chief physician	11–15	5–10	101–200

**Table 4:** Themes and sub-themes on the influencing factors of transition

Themes	Sub-themes	Facilitator(s)	Barrier(s)
**Intra-individual Factors**			
Personal characteristics	Personal characteristics of patients	Old; prolonged disease duration; mild disease	Young; newly diagnosed; severe disease; academic pressures such as the Gaokao*
	Personal characteristics of parents	Low education level; busy work	High education level
Disease acceptance	Disease acceptance by patients	High level of disease acceptance	Low level of disease acceptance
	Disease acceptance by parents	High level of disease acceptance	Low level of disease acceptance
Transition consciousness	Aware of the necessity for transition	√	Lack of awareness of the necessity for transition
	Aware of patient autonomy	√	Lack of awareness of patient autonomy
Mentality of guilt	Mentality of guilt experienced by patients	√	/
	Mentality of guilt experienced by parents	/	√
Self-efficacy	Self-efficacy in transition by patients	High level of self-efficacy	Low level of self-efficacy
	Self-efficacy in transition by providers	High level of self-efficacy	Low level of self-efficacy
**Intra-individual Factors**			
Parent-child separation	Geographical separation of residence	√	/
	Psychological separation of attachment	√	/
Transition communication	Lack of communication	/	√
	Tough attitude	/	√
	Educating patiently	√	/
**Macro-environmental Factors**			
Community support	Patient peers	√	/
	The China Crohn’s & Colitis Foundation	√	/
Healthcare environment and policies	The current state of the Chinese transition environment	/	√
	Medical policy for minors	/	√
	Hospital guide services	√	/

*Gaokao is the College Entrance Examination in China;√indicates that the factor associated with the given row pertains to the contents of the corresponding column; / indicates not applicable

**Fig. 1:**
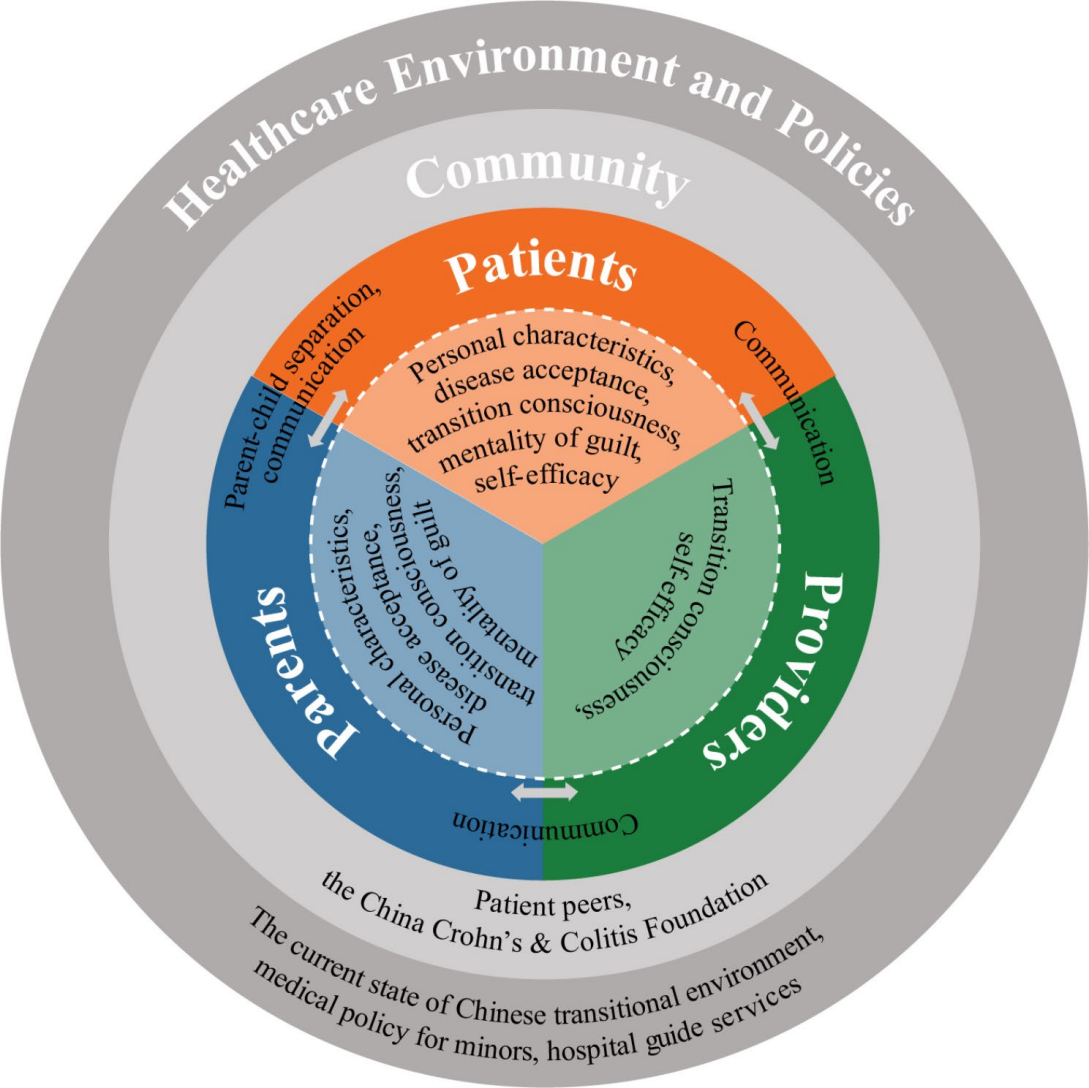
Depiction of the thematic structure involving all stakeholder groups

Based on our findings, we constructed a framework depicting the factors influencing the transition of Chinese adolescents with IBD, as shown in Fig. 1. The analysis reveals that the transition process is shaped by interactions within and between individuals, including patients, parents, and healthcare providers, as well as by macro-environmental factors.

### Intra-individual factors

#### Theme 1: personal characteristics

##### Personal characteristics of patients

The personal characteristics of patients, such as age, disease duration, disease severity, and academic phase were significant. Younger patients or those with newly diagnosed or severe disease often have their parents closely involved in managing their condition. The academic phase, particularly the Gaokao period, demands considerable attention, leading to a focus on studies rather than transition planning. *“In senior school, I did not have that much time for medical appointments, diet management, or anything else. My parents took care of my disease a little bit more during that time.”* (A1).

##### Personal characteristics of parents

The educational level and work demands of parents influence the transition. Parents with limited education may struggle to understand the disease, which can push patients to take more responsibility for their condition. Conversely, more educated parents tend to be more involved in managing their children’s disease. *“Those educated parents, some of them would do everything for their children. For example, there was a child in his 20s whose parent still has full authority to manage the disease for him.”* (C5).

When parents have demanding work schedules, patients often take on more responsibility for their medical care. *“My parents did not have the time to manage my medication when they went out to work. Thus, I had to take my medication independently.”* (A12).

#### Theme 2: disease acceptance

##### Disease acceptance by patients

During the early stages of diagnosis, patients often resisted accepting their disease and experienced feelings of confusion, questioning their situation with “Why me?” sentiments. This reluctance to accept their condition can impede the transition. For instance, a parent recounted, *“Once, my child cried loudly and said, ‘Why am I the only kid who can’t eat?’ He couldn’t understand why he was the only unlucky one with this disease among so many children. And he always resisted going to the follow-up appointment.”* (B5).

Acceptance of their illness is a critical foundation for adolescents’ transition. Discussing transition prematurely, before acceptance, can be detrimental. As a parent noted, *“At the time of the initial diagnosis, he was quite sad, probably because I spoke too much about the transition and spoke too early. Later I knew that he had to accept his disease before we could move forward.”* (B6).

##### Disease acceptance by parents

Most parents experienced anxiety after their children were diagnosed and became deeply involved in their children’s care, which may delay the transition. As parents come to terms with the illness, they can then contemplate the transition. *“At that time, the parents were very anxious, they became too overly involved in the management of their children’s disease. It was not an appropriate time to discuss transition”* (C1).

#### Theme 3: transition consciousness

##### Aware of the necessity for transition

The consciousness of the necessity of transition should be established among stakeholders. Some patients may initially not realize the necessity of transitioning and resist taking charge of their disease management. When patients and their parents realize that IBD is a lifelong disease, they understand that independent management of the disease is necessary. One patient expressed, *“Later, when I knew that the disease would take a long, long time to treat, I wanted to stop relying on anyone.”* (A9).

Many healthcare providers overlook the importance of a structured transition for adolescents with IBD, viewing it as a natural progression and not prioritizing it. This lack of focus on transition can be a significant obstacle. *“Children will naturally transition to adult hospitals when they grow up, and we usually do not pay much attention to this, it is not our main focus.”* (C8).

##### Aware of patient autonomy

During the transition, patients’ growing consciousness of their physical and medical conditions equips them for better disease management. One patient shared, *“I believe that I should take a leading role in disease management, from scheduling medical appointments to managing medication and paying medical bills. Moreover, actively engaging with my doctor has been helpful for me.”* (A1).

Some parents realized that the patient should play a principal role in disease management, which encouraged them to begin exercising and guiding their children in transition. Providers also played an important role in educating parents regarding the need for patient autonomy. *“We advised parents that holding onto their children would impede the children’s growth towards independence. They could not keep holding on, or they would have a ‘giant baby’.”* (C5).

#### Theme 4: mentality of guilt

##### Mentality of guilt experienced by patients

Many patients felt guilty about the financial and emotional strain their condition placed on their parents, driving them to manage their disease independently. *“I feel very sorry for them. Since I have no income, I rely on them for financial support. I manage my disease to reduce their burden.”* (A9).

##### Mentality of guilt experienced by parents

Parents also grappled with guilt over their child’s diagnosis, often feeling compelled to provide extensive support. However, such a mindset of parents can hinder this transition. *“I felt very guilty. When I saw other children healthy and my child sick, I felt sorry for him. I just wanted to accompany him and help him as much as I could.”* (B5).

#### Theme 5: self-efficacy

##### Self-efficacy in transition by patients

Self-efficacy, or patients’ confidence in their ability to manage their transition, is crucial. Some patients with low self-efficacy believed that they were incapable of managing their condition well, leading them to rely more on their parents. *“I didn’t do anything. My father took me to the hospital and managed everything. I wouldn’t do it, and I wouldn’t be able to do it.”* (A11).

Conversely, supportive parents and providers can foster independence and enhance self-efficacy. *“My mother drew a flow chart of what I needed to do. After memorizing that chart, I could go to the hospital by myself.”* (A6).

##### Self-efficacy in transition by providers

Healthcare providers’ self-efficacy pertains to their confidence in facilitating patient transition. However, many providers doubt their influence, feeling that family education is the decisive factor in transition, a domain they cannot significantly alter. This belief could constrain the providers’ guidance in the transition process.*“In the past ten or twenty minutes, can you change his family education in the past ten years? I think it is impossible.”* (C4).

### Inter-individual factors

#### Theme 6: parent-child separation

##### Geographical separation of residence

For some families, changes in living arrangements due to work or other factors have led to geographical separation, inadvertently advancing the transition by reducing parental involvement and promoting patient independence. *“When I went to college, I was too far away from home to go to the hospital with my mother. This geographical isolation forced such a situation (transition).”* (A10).

##### Psychological separation of attachment

The psychological bond between patients and parents can create a dependency, with patients seeking comfort from their parents in managing their condition. *“I can bear my disease, but they must accompany me, by my side. It will make me feel safe.”* (A6).

#### Theme 7: transition communication

##### Lack of communication

Communication barriers often stem from patients’ reluctance to discuss their condition, leading to silence even when parents inquire. *“I don’t like talking to my parents very much. My mother kept asking me about my disease, but I kept quiet because I didn’t like talking to her.”* (A1).

##### Tough attitude

Some parents communicate with a tough attitude during the transition, which may not only be ineffective but can also cause friction. *“They exhibited a tough attitude and told me to manage my disease more, without considering how I felt. I did not like the way they asked me to do it.”* (A3).

##### Educating patiently

Most healthcare providers believed that educating patiently was essential for a successful transition by ensuring patients have the latest disease-related knowledge. *“I would tell the children the latest information about the disease, including diet and drug information.”* (C10).

Additionally, when the providers identified inappropriate disease management behaviors in patients, they corrected them promptly to help them transition successfully. *“There was a doctor who rebuked my intermittent medication behavior, and corrected me seriously.”* (A7).

### Macro-environmental factors

#### Theme 8: community support

##### Patient peers

The sharing of experiences among patient peers is beneficial in supporting adolescents who need to transition. It can encourage their participation in a smoother transition process. *“She took the initiative to communicate with her patient peers on how to manage her disease. Now the disease is all managed by her, and she has changed a lot.”* (B3).

Furthermore, peers could provide psychological support, which was vital for helping adolescent patients manage their emotions related to the disease and approach the transition with a more positive outlook. *“They are all suffering from this disease, so I can sympathetically communicate with them and gain a little comfort from them, at least the pressure is not as heavy as the first contact.”* (A7).

##### The China Crohn’s & Colitis Foundation

The China Crohn’s & Colitis Foundation is a public welfare organization dedicated to improving the quality of medical treatment and life of patients with IBD [28]. It plays a crucial role in helping adolescent patients and their parents understand the disease, adjust their mental state, and facilitate the transition. This support is primarily through organizing summer camps and disseminating information via social media. *“There was psychological counseling in the summer camp. Since then, I began to accept this disease, and then felt that I should manage this disease by myself.”* (A6).

#### Theme 9: healthcare environment and policies

##### The current state of the Chinese transitional environment

In China, there is a distinct separation in health education between adults and minors, leading to a gap where adolescent patients often do not receive systematic transitional education. *“There’s no content on transition in health education right now; no one ever talks to them about how to transition.”* (C7).

Additionally, there is no direct parallel referral between the pediatric and adult healthcare systems. The pediatric system provides a discharge summary, printed endoscopic images, and other written medical records, but the transmission of comprehensive disease information is not guaranteed. *“Doctors in our children’s hospital would write a summary of the whole treatment and send the printed endoscopic images to the parents. However, there must have been problems with the reporting of transition or keeping paper records.”* (C10).

##### Medical policy for minors

In China, hospital policies dictate that minors cannot assume legal responsibility, and all medical decisions must be authorized by their guardians. This policy necessitates parental presence, thereby restricting minors’ opportunities for independent treatment. *“All the signatures in the children’s hospital have to be done by parents, and it was useless for the children to go by themselves.”* (B6).

##### Hospital guide service

Hospital guide services facilitate independent treatment for adolescent patients. For instance, with biological therapy, the hospital staff provided detailed flowcharts outlining the process. *“As long as you go by yourself, there are a lot of people who will guide you on what to do next.”* (A10).

## Discussion

This study represents one of the pioneering efforts in China to assess the factors affecting the transition of adolescent patients with IBD, incorporating the perspectives of patients, parents, and healthcare providers. Our findings partially corroborate the SMART model’s results [20, 21]. Nevertheless, our study identifies unique facilitators and barriers within the specific context of China’s national conditions and cultural backgrounds.

The research suggests that older patients tend to navigate the transition process more effectively, aligning with previous studies [29, 30]. This correlation may be attributed to the development of cognitive functions and self-management skills with age. A longer duration of the disease also appears to ease the transition, likely due to the patient’s deeper understanding and management experience. Skills crucial for a successful transition, such as disease-specific knowledge, self-efficacy, and decision-making [9], are honed through ongoing disease management. Interestingly, one study suggested that a prolonged disease duration correlates with a less significant improvement in coping with IBD, implying that coping skills may improve incrementally over time [31]. Our analysis also indicates that stable disease conditions are vital for patient transition, which is in line with expert consensus [9]. However, there is conflicting evidence about the relationship between disease activity and readiness for transition [32]. While some studies have highlighted better transition outcomes for females than males [29, 30], this study found insufficient evidence to determine gender’s impact on the transition.

The academic stage was a unique factor that influenced the transition in this study. In China, the college entrance examination, or “Gaokao,” is one of the most important talent selection systems and the primary pathway to higher education for high school graduates [33]. Unlike the more flexible college admissions approach of American students, Chinese students often devote the majority of their high school years to Gaokao preparation [34]. Therefore, adolescent patients with IBD are more inclined to prioritize Gaokao preparation over managing their disease transition. It is logical to consider Gaokao as a factor that may delay the transition.

The study also identified the mentality of guilt by patients as an influential factor. This guilt often stems from their perception of not fulfilling the expectations of their parents, who have invested time, money, and emotional support in their upbringing and education. Research indicates that Chinese students recall guilt/shame-related events more frequently than American students (23.96% vs. 5.77%), with a notable focus on academic performance and meeting parental expectations [35]. Although typically viewed as a negative emotion, guilt can motivate patients towards self-management and transition. Rooted in Confucian thought [36], Chinese students may be more likely to transform feelings of guilt into a positive, redemptive action, such as actively participating in disease management. Hence, when adolescents with IBD feel guilt, it’s important for their parents and healthcare providers to help them channel this emotion constructively, utilizing it as a catalyst for transition.

Parents play a crucial role in the transition of adolescent patients with IBD as they spend the most amount of time with their children. Parents’ educational level, which had a dual effect, was another factor affecting transition. In our study, parents with higher education levels often took an excessive role in their children’s disease management. Conversely, those with lower education levels, while slower to grasp IBD knowledge and engage with healthcare providers, inadvertently pushed their children towards independent disease management. This finding contrasts with the cultural factors in the SMART model [20]. While much of the existing research focuses on the impact of patients’ educational levels on transition [32, 37], the influence of parents’ education on the process is less explored. The SMART model suggests that a cooperative relationship between patients and their parents is conducive to a smooth transition [20]. However, such a relationship is uncommon in China. Chinese parents tend to adopt a didactic and relationship-oriented parenting style [36]. In our study, some parents’ authoritarian and tough communication approach added to the psychological burden of patients, incited conflicts, and led to resistance against transition. Notably, the adolescent transition period is already marked by emotional turbulence and a tendency to challenge parental authority [38]. Such a tough stance in communication can exacerbate conflicts, thereby obstructing the transition process. Furthermore, neglect of transition guidance and parent-child communication can leave patients ill-prepared for independent disease management, leading to negative transition outcomes. Parent-child discussions about the transition can enhance patients’ self-management abilities [39].

The Chinese cultural norm involves significant parental involvement [40]. However, this study illustrates that excessive involvement may not always aid disease management. Firstly, parents may not recognize changes in the disease promptly, potentially delaying treatment. Secondly, it can lead to patient dependency, complicating future separation. Additionally, some patients reported that their parents showed excessive concern about the disease, with conversations limited to physical symptoms while ignoring their adolescent experiences and psychological well-being. Parents should be encouraged to learn to balance the quantity (i.e., not excessive) and quality (i.e., not limited to the disease) of involvement, particularly during the sensitive and vulnerable adolescent years of their children.

The theme of “parent-child separation” in this study encompasses both geographical and psychological aspects. The former is tangible and less changeable, while the latter is intangible and more amenable to adjustment. The cultural context of East Asia tends to view the self as “interdependent” or “collectivist,” in contrast to Western societies that value “independence” or “individualism” [36]. According to traditional and empirical data, the Chinese self is generally considered to have a dominant orientation of interdependence and relatedness [36]. Moreover, under the enduring influence of Confucian family values, the Chinese show the greatest interdependence and emphasis on kinship [36]. Therefore, it seems understandable that most adolescent patients with IBD in this study had difficulty achieving parent-child separation by excessively relying on their parents. However, some of the main goals of adolescents are to achieve autonomy, become less dependent on parents, and gain greater responsibility [41]. It is difficult for parents to strike a balance between controlling and letting go [42]. Transferring responsibility is not simple for any parent, although it is often taken for granted. Parental training that focuses on adapting to changing family roles can provide valuable support for adolescents in their transition to independence [43].

This study highlighted that some healthcare providers demonstrated a lack of awareness and self-efficacy in managing the transition of patients with IBD. This attitude predominantly stems from a belief that the onus of transition rests with the parents and that providers have little power to influence the outcomes of long-standing family education. Additionally, the limited outpatient time for physicians in China, dictated by a performance evaluation system linked to service volume and workload, further complicates this issue [44]. With most Chinese outpatient physicians already facing moderate to high levels of task and mental workload [45], integrating patient transition management into the brief outpatient interactions is challenging. A significant number of primary healthcare physicians feel unequipped to manage IBD patients [46]. Yet, healthcare providers who are proactive and confident in their approach can facilitate a smoother transition [47]. They can cover various transition-related topics with adolescents, such as IBD knowledge, medical history, medication, tests, independence, self-efficacy in medication management and clinic appointments, lifestyle choices [48, 49]. In addition, meeting with a new physician and building trust with healthcare providers are common concerns of patients during this transition [50].

The transition within healthcare systems faces obstacles like the absence of a formalized transition protocol, patients’ unreadiness for transition, and the lack of trust in adult healthcare providers [51]. This is corroborated by surveys indicating that adult gastroenterology providers often receive insufficient medical history from pediatric counterparts [52], aligning with our findings. The most commonly endorsed models for IBD transition recommend collaboration between pediatric and adult IBD teams, including gastroenterologists and IBD nurse specialists [9]. The role of a transition process coordinator, typically a pediatric IBD nurse, is considered crucial [9]. A joint pediatric-adult clinic model is seen as ideal within a transition program [9]. While there is no definitive evidence pointing to the superior efficacy of any one model, the components that have been identified as beneficial can be used as a reference.

This study has identified factors affecting the transition of adolescent patients with IBD from the perspectives of patients, parents, and healthcare providers. We recommend that adolescents should assert their independence in managing their disease, reduce reliance on parents, and take proactive charge of their health, while balancing academic demands and health needs. Parents need to mitigate feelings of guilt, refrain from over-involvement, and prioritize effective communication during the transition. Healthcare providers are urged to focus on the transition process and bolster the acceptance and self-efficacy of adolescents and their parents through education. They should also facilitate connections with peer support or public welfare organizations for additional social backing. The development of structured transition programs is critical to ensure continuous care for adolescents with IBD. Provider training on transition-related issues appears imperative.

## Limitations

The interpretation of this study’s findings is subject to several limitations. The sample’s limited diversity might affect the broad applicability of the findings. The participants were all from the same set of healthcare centers, potentially reflecting the experiences of adolescents more engaged in outpatient care management for IBD. Additionally, the necessity for online interviews with 14 participants due to the COVID-19 pandemic could have impeded the interviewer’s ability to observe non-verbal cues as effectively as in-person interviews.

## Conclusions

Chinese adolescents with IBD face unique challenges during their transition. It is imperative for patients, parents, and healthcare providers—as the primary stakeholders—to recognize and address the facilitators and barriers within the transition process, aiding in the movement toward successful independence. While an ideal IBD transition program has yet to be established, further research should aim to enhance transition strategies considering the perspectives of each stakeholder involved.

## Data Availability

The data that support the findings of this study are available from the corresponding author upon reasonable request.

## Abbreviations

CD: Crohn’s disease
IBD: Inflammatory bowel disease
UC: Ulcerative colitis
SMART: Social-ecological model of adolescents and young adults readiness to transition
SRQR: Standards for Reporting Qualitative Research
